# Two-in-one system and behavior-specific brain synchrony during goal-free cooperative creation: an analytical approach combining automated behavioral classification and the event-related generalized linear model

**DOI:** 10.1117/1.NPh.10.1.013511

**Published:** 2023-02-10

**Authors:** Mingdi Xu, Satoshi Morimoto, Eiichi Hoshino, Kenji Suzuki, Yasuyo Minagawa

**Affiliations:** a Keio University, Center for Life-span Development of Communication Skills, Yokohama, Japan; b Keio University, Global Research Institute, Tokyo, Japan; c University of Tsukuba, Faculty of Engineering, Information and Systems, Tsukuba, Japan; d Keio University, Department of Psychology, Faculty of Letters, Tokyo, Japan

**Keywords:** functional near-infrared spectroscopy hyperscanning, automated behavior classification, two-in-one system, superior temporal gyrus, temporo-parietal junction

## Abstract

**Significance:**

In hyperscanning studies of natural social interactions, behavioral coding is usually necessary to extract brain synchronizations specific to a particular behavior. The more natural the task is, the heavier the coding effort is. We propose an analytical approach to resolve this dilemma, providing insights and avenues for future work in interactive social neuroscience.

**Aim:**

The objective is to solve the laborious coding problem for naturalistic hyperscanning by proposing a convenient analytical approach and to uncover brain synchronization mechanisms related to human cooperative behavior when the ultimate goal is highly free and creative.

**Approach:**

This functional near-infrared spectroscopy hyperscanning study challenged a cooperative goal-free creative game in which dyads can communicate freely without time constraints and developed an analytical approach that combines automated behavior classification (computer vision) with a generalized linear model (GLM) in an event-related manner. Thirty-nine dyads participated in this study.

**Results:**

Conventional wavelet-transformed coherence (WTC) analysis showed that joint play induced robust between-brain synchronization (BBS) among the hub-like superior and middle temporal regions and the frontopolar and dorsomedial/dorsolateral prefrontal cortex (PFC) in the right hemisphere, in contrast to sparse within-brain synchronization (WBS). Contrarily, similar regions within a single brain showed strong WBS with similar connection patterns during independent play. These findings indicate a two-in-one system for performing creative problem-solving tasks. Further, WTC-GLM analysis combined with computer vision successfully extracted BBS, which was specific to the events when one of the participants raised his/her face to the other. This brain-to-brain synchrony between the right dorsolateral PFC and the right temporo-parietal junction suggests joint functioning of these areas when mentalization is necessary under situations with restricted social signals.

**Conclusions:**

Our proposed analytical approach combining computer vision and WTC-GLM can be applied to extract inter-brain synchrony associated with social behaviors of interest.

## 1 Introduction

Investigating a single human brain is insufficient for fully understanding the neural mechanisms underlying human social behaviors as the human social brain does not merely observe social stimuli, but interacts in social encounters. Second-person neuroscience considers the dynamic interplay between multiple brains and has become a burgeoning field of interest.^1^^–^^5^ Hyperscanning techniques using various measurement modalities have been used to study the neural correlates of multiple individuals engaged in social interactions. Due to its high flexibility and desirable spatial and moderate temporal resolution, functional near-infrared spectroscopy (fNIRS) hyperscanning plays a significant role in the field of naturalistic imaging.

Early fNIRS hyperscanning studies adopted experimentally controlled tasks that may not be sufficiently naturalistic, such as simultaneous motor action,^6^ motor imitation,^7^ and coordinated speech rhythm.^8^ In these tasks, two participants were required to act synchronously; thus, the observed brain synchronization could be interpreted as partially related to simultaneous motor movements. More recent studies have used natural or free settings such as nonstructured verbal communication,^9^^,^^10^ eye-to-eye contact,^11^^,^^12^ turn-taking games,^13^ and group creativity.^14^ Because the target behaviors of these experiments were largely unscheduled, coding works were usually necessary to disentangle the relationship between the target behavior and particular brain synchronization. This raises the following dilemma: as tasks are made more naturalistic, more time-consuming coding is necessary. However, this is not an insignificant issue. Such an analysis is acceptable for short-term social interactions, but it is difficult for longer ones. Human-interactive behaviors may not necessarily be short, and naturalistic behaviors should be captured free from such time restrictions.

This study addresses this problem by proposing an analytical approach for naturalistic fNIRS hyperscanning. This methodological consideration was the first objective of this study. We used “computer vision” for automated behavior estimation from video-recordings. To examine the relationship between automatically extracted behaviors and particular neuronal synchrony, a generalized linear model (GLM) was applied in an event-related manner. This method can estimate which social signal correlates with which type of brain synchronization, in addition to the ordinarily performed condition-based analysis.

The second objective of this study was to uncover brain synchronization mechanisms related to human cooperative behavior when the ultimate goal of cooperation is highly free and creative. The cooperative tasks used in previous fNIRS hyperscanning studies generally have in common that they have relatively fixed goals that are known in advance, such as pressing a button at the same time as much as possible or completing a Jenga game together.^13^ For such tasks, as long as one’s behavior does not deviate significantly from the task instruction, one does not need to mentalize the partner’s thinking moment by moment. However, real-life cooperation is full of complex cases, such as situations in which the ultimate goal of communication is highly variable, depending on the ever-changing behavior and mental state of the communicating partners. Such situations require communicating parties to continuously mentalize the intentions of their partners and adjust their own behaviors accordingly.

This study designed a free-style joint creative computer game to capture dynamic aspects of naturalistic social interaction. Two players were asked to design the interior of a room that satisfied both parties or just themselves, without limitations on time or communication. The two conditions (cooperative and independent) were compared to reveal between- and within-brain synchronized (WBS) activities specific to each condition. Furthermore, from global brain synchronizations elicited by cooperative creation, we attempted to extract those corresponding to particular social behaviors (e.g., face-to-face). The facial orientation of a participant, roughly comparable to the presence/absence of eye gaze, contains active social cues to the partner.^11^^,^^15^^–^^20^ Consequently, our study used the face orientation of dyads as GLM regressors to isolate correlated brain synchronizations. Synchronizations in the brain regions of the mentalizing network^9^^,^^11^^,^^13^^,^^14^^,^^21^^–^^23^ and mirror neuron system^16^^,^^20^^,^^24^^,^^25^ are expected to be related to face-to-face behaviors in our study.

Our study proposes an analytical approach without manual behavioral coding to extract the neural correlates of targeted social signals (face-to-face). With this approach, we sought to uncover behavior-specific brain synchronizations among various interactive social behaviors and neural synchrony elicited by a task demanding collaborative creation without time constraints.

## 2 Methods

### 2.1 Participants

A total of 78 college students [54 females; 18–32 years: mean ± standard deviation (SD) = 19.8 ± 2 years] participated in the experiment. This resulted in 39 same-sex dyads (27 female dyads). Five participants were left handed. None of the participants reported sensory, neurological, or psychiatric disorders. Written informed consent was obtained from all participants following the Declaration of Helsinki. The experimental protocol was approved by the Ethics Board of Keio University, Japan (No.16028).

### 2.2 Tasks and Procedures

**Fig. 1:**
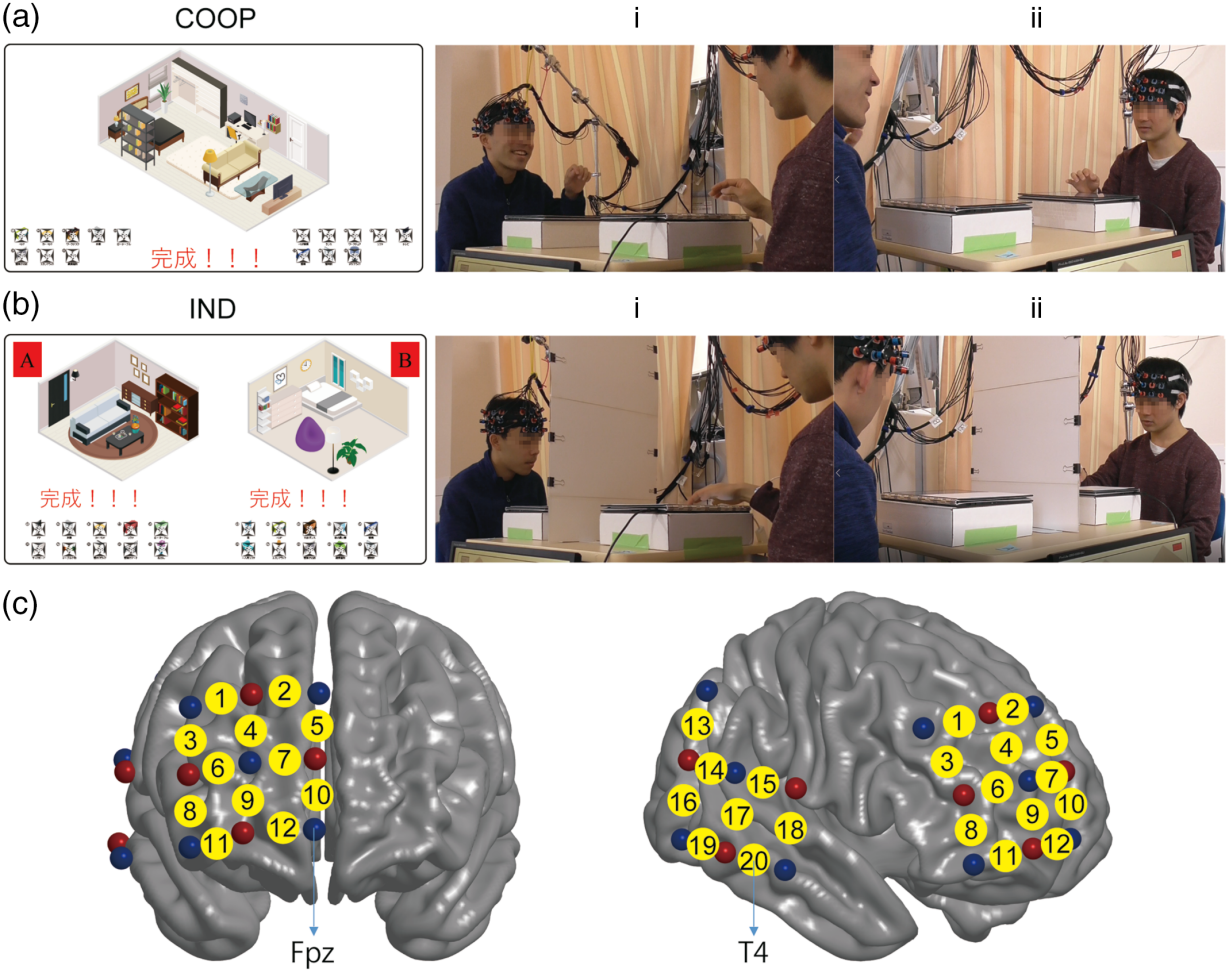
Experimental design. (a) and (b) Experimental images for the cooperative (COOP) and independent (IND) tasks, respectively. Left: completed interior design for a sample dyad. Right: videos recorded for a sample dyad. (i) Participant A and (ii) Participant B. (c) Probe set and channel layout (20 in total) in the PFC and temporal region of the right hemisphere. Red dots: emitters; blue dots: detectors; and yellow dots: channels. Brain images were created using BrainNetViewer.^26^

**Fig. 2:**
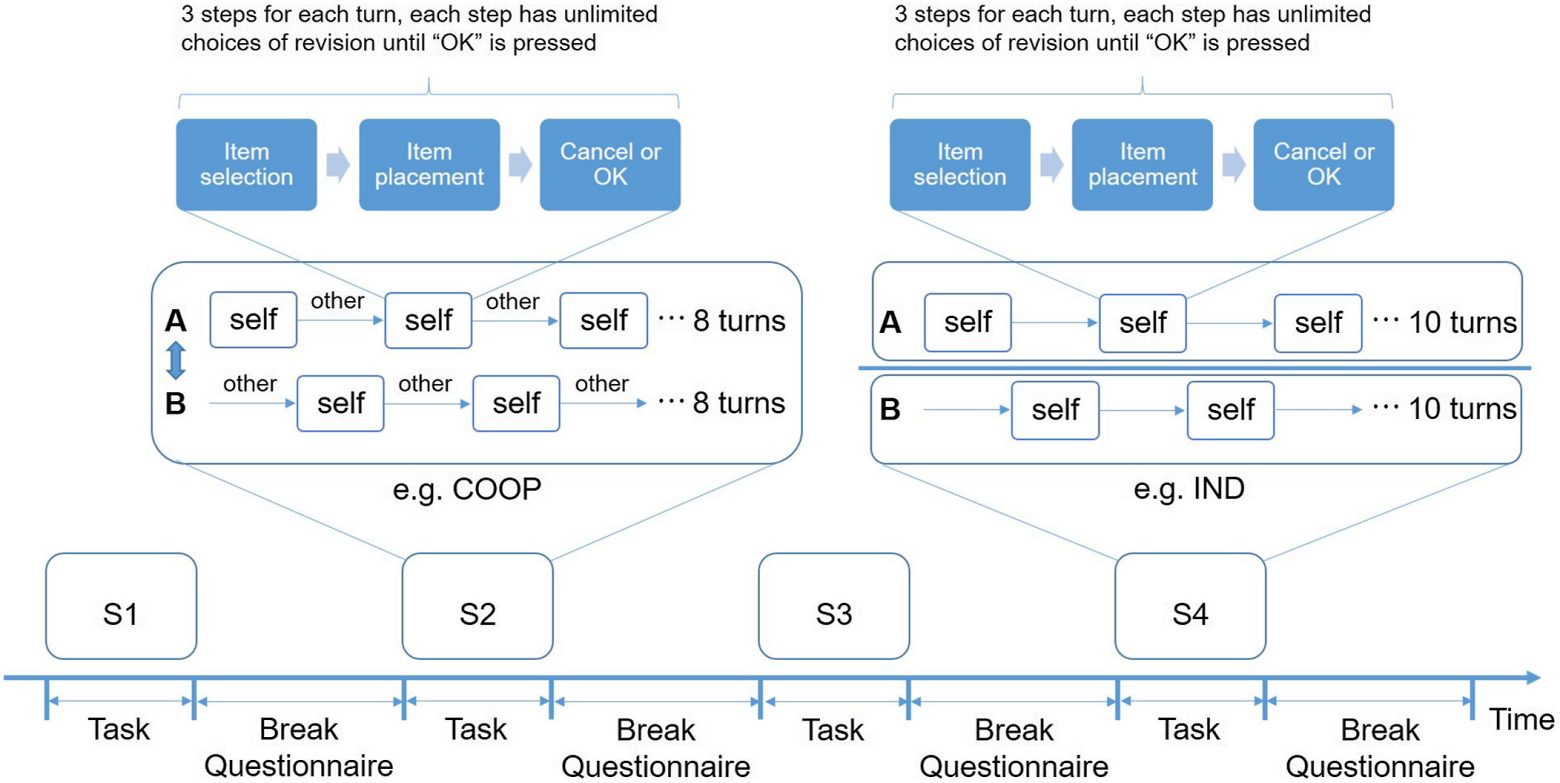
Experimental paradigm for the turn-based computer game. S1–S4: four counterbalanced sessions that include two cooperative (COOP, e.g., S2) and two independent (IND, e.g., S4) conditions. During breaks, participants answered a simple questionnaire and had a rest until they were ready for the next session (no time limit and mostly longer than 3 min).

We designed a turn-based interior design browser game that ran on a server (Sec. 1.1 in the Supplementary Material). The dyad sat face-to-face and manipulated their touch panels [Fig. 1(a) and Fig. 1(b)]. Each dyad completed two sessions of cooperative game (COOP) and two sessions of independent game (IND) throughout the experiment. The order of the four sessions was counterbalanced among dyads (Fig. 2). Before fNIRS recording, each dyad practiced (communication allowed) until they fully understood the game procedure and rules. They were informed that three referees would rate their rooms to increase their motivation.

In the COOP condition [Fig. 1(a)], participants were asked to furnish a room that satisfies not only themselves but also their partner in a turn-based manner (Fig. S1 in the Supplementary Material). In one turn, only the on-duty participant was allowed to manipulate the touch panel, while the partner could express their opinions. They were encouraged to communicate freely and as much as necessary to reach a consensus on each item. Each COOP session took ∼11.5  min (SD = 9.6). In the IND condition, each participant was asked to furnish a room by himself/herself. A partition board was placed between them to prevent them from seeing each other [Fig. 1(b)]. Each IND session took ∼9.6  min (SD = 4.1).

Two video cameras (Panasonic, HC-V360M, Full HD, 29.97 fps) were used to record the entire experiment. Each camera was focused solely on one participant (Sec. 1.2 in the Supplementary Material). After each game session, participants completed a task questionnaire (enquiring about the degree of satisfaction, cooperativeness, concentration, etc.). No discussions were allowed.

### 2.3 fNIRS Data Acquisition

An optical topography system (OT-R40, Hitachi Medical Company, Japan) was used to collect imaging data from the two participants in each dyad simultaneously. The absorption of near-infrared light at two wavelengths (695 and 830 nm) was recorded at a sampling rate of 10 Hz.

Two identical sets of customized probe arrays consisting of two separate pads were used for each dyad to simultaneously record brain activity from the right temporal and prefrontal regions, which has shown to be robustly involved in social interactions.^14^^,^^22^ Each participant had 20 channels (Fig. 1(c); Sec.1.3 in the Supplementary Material). The virtual registration method^27^^,^^28^ was used to estimate the brain area covered by each channel according to automatic anatomical labeling or Brodmann area (Chris Rorden’s MRIcro).^29^ All probes were adjusted carefully using the international 10–20 system to ensure consistency of the positions on the head across participants to apply the virtual registration.

### 2.4 fNIRS Data Analysis

Changes in oxygenated hemoglobin (oxy-Hb) and deoxygenated hemoglobin (deoxy-Hb) concentrations were calculated for each channel using the modified Beer–Lambert law. Because temporally synchronized motion would cause some artifacts to brain synchronization, we carefully applied two types of statistical methods for pre-processing. The hemodynamic modality separation (HDMS) method^30^ separates the cortical hemodynamic change from systemic hemodynamic signals (e.g., skin-blood flow) based on their difference in statistical properties due to oxygen metabolism. Therefore, HDMS removes superficial blood signals derived from interactive movements (e.g., nodding and talking). The pre-whitening procedure^31^ removes serial correlation in the signal, so motion artifacts could also be eliminated statistically. The oxy-Hb and deoxy-Hb data were filtered using an AR-model-based pre-whitening filter. The order of the AR filter was set to 50 (i.e., 5 s). The wavelet transformed coherence (WTC)^32^ was applied to the time series of oxy-Hb and deoxy-Hb from two participants of a dyad to evaluate the cross-correlation between their brain signals as a function of time and frequency.^6^ WTC analysis was performed for each possible channel-pair (210 in total), including both identical-channel-pair (20) and different-channel-pair (190) (Sec. 1.4 in the Supplementary Material). We used a Morlet wavelet (with the wavenumber $\Omega_{0}=6$) as the mother wavelet, which provides a good tradeoff between time and frequency localization.^32^ Consequently, there were 91 frequency bands at 1/10 octave intervals (0.0072–3.68 Hz) for each channel-pair. We calculated the mean value of the WTC time series for each channel-pair in each frequency band, resulting in 210×91=19,110 channel-channel-frequency (ch-ch-fr) combinations of the mean WTC for each dyad. For this calculation, we ignored values outside the cone of influence to remove boundary effects.

To investigate between-brain synchronization (BBS), we compared the mean WTC between COOP and IND conditions. A two-tailed t-test was applied to each ch-ch-fr. Due to the large number of ch-ch-fr, controlling the family-wise error rate to reduce the type 1 error (e.g., Bonferroni’s correction) would produce too many false negatives. Therefore, we practically controlled the false discovery rate (FDR) using Storey’s procedure^33^ (Sec. 1.5 in the Supplementary Material). The WTC within each single brain (WBS, or functional connectivity) was also calculated for each ch-ch-fr. There are 190×91=17,290 ch-ch-fr for the WBS. The same statistical methods and criteria as in the BBS were used.

### 2.5 Behavioral Data Analysis

**Fig. 3:**
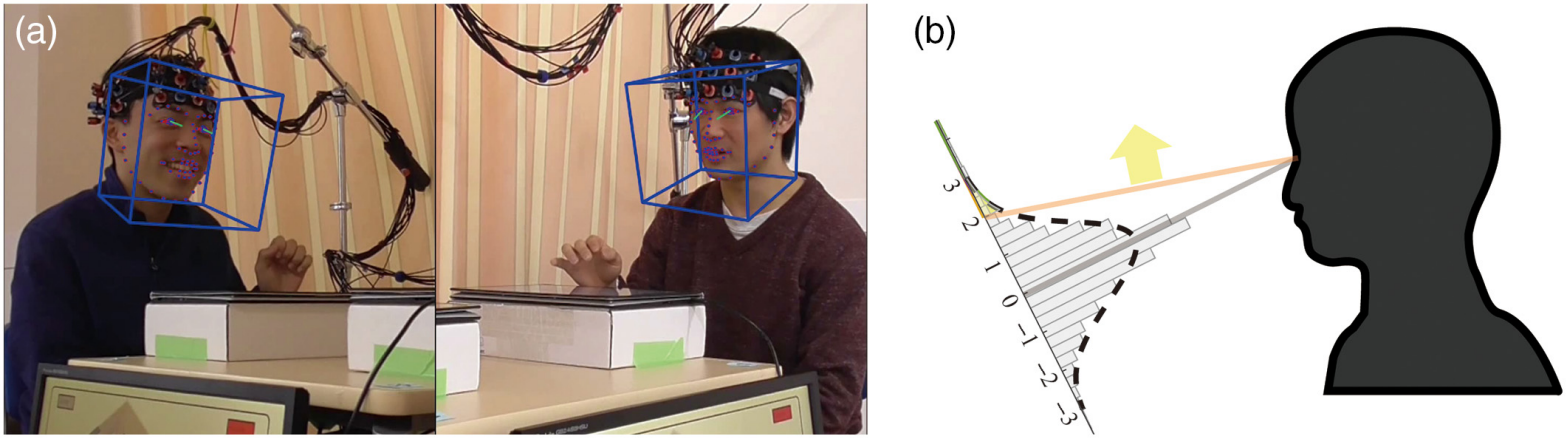
Automated detection of face-up events using OpenFace 2.1.0., which can automatically detect facial landmarks for every frame and track face orientation from video sequences. (a) Illustration of the face landmarks (colored dots) and face orientations (blue cubes) of a sample dyad. (b) Illustration of the threshold (2 SD) used to detect face-up events. The axis represents the z-score of the pitch angle.

OpenFace 2.1.0,^34^ an open-source toolbox for MATLAB (Mathworks Inc.), was used to estimate face orientations of the dyads in the two COOP sessions from video sequences [Fig. 3(a)]. The face orientation was estimated as rotation in world coordinates with the camera serving as the origin and representing pitch, yaw, and roll form in radians (Sec. 1.6 and Fig. S2 in the Supplementary Material). We focused on the pitch angle and regarded it as the parameter for evaluating face-up events, which can be considered as salient social behavior, roughly corresponding to eye gaze.

The pitch angle was transformed into a z-score to ignore the difference between the heights of the two faces. We used 2SD of the z-scored pitch angle as the threshold to classify a face-up event [Fig. 3(b); and Fig. S2 in the Supplementary Material]. This strict classification threshold selected ∼2.3% of the video sequence as the face-up state. We verified (visually inspected) that, during these “face-up” events, participants raised their faces from their touch panels and socially engaged with their partners.

Face-up events were labeled in the two COOP sessions (Fig. S2 in the Supplementary Material) to be used as regressors in the following GLM analysis. This procedure extracted the temporal information of (1) when both participants raised face (“both-up”) and (2) when only one participant (e.g., participant A) raised face (“self-up” for participant A and “other-up” for participant B).

### 2.6 Assessment of WTC Correlates of Social Behaviors

The GLM is a flexible model of multivariate linear regression in which the response variable is allowed to follow a nonnormally distributed error distribution. We used a GLM to examine the relationships between WTC and targeted interactive behaviors (here, face-up events) in an event-related manner. WTCs that showed significantly larger values (p<0.001) in COOP than in IND in the frequency bands range of 0.03–0.1 Hz (Sec. 1.5 in the Supplementary Material) were used as dependent/response variables. The WTC of one representative frequency band (which showed the smallest p-value) was selected for each ch-ch-fr as WTCs from adjacent frequency bands were highly correlated.

For the WTC-GLM analysis (Sec. 1.7 in the Supplementary Material), the time courses of the aforementioned three types of face-up events serving as regressors were linked to the time course of the WTC for each representative ch-ch-fr. First, the time courses of the behavioral regressors (X) were down-sampled to 10 Hz to correspond to that of the WTC (Y). The WTC was adjusted for the delay-of-peak effect (5 s). Second, for each representative ch-ch-fr, we estimated the coefficient β to minimize the error ε:

Y=βX+ε.

Y: WTC matrix

X: design matrix (both-up, self-up, other-up, and constant; four regressors).

For the distribution of the error ε and link function in GLM, we used a gamma distribution and log-link function, respectively. Third, the βs for “both-up” (identical for participants A and B) and “either-up” (self-up of participant A is identical to other-up of participant B, and vice versa, so they were combined as either-up) regressors in each ch-ch-fr of interest (WTC: COOP > IND) were compared using paired t-tests (p<0.05, Bonferroni’s correction).

## 3 Results

We will report our oxy-Hb results here and provide relevant information about the deoxy-Hb data in Fig. S3 in the Supplementary Material. The results (synchronized channel-pairs) of the deoxy-Hb were nearly the same as that of oxy-Hb with some differences in statistical values (Tables S1–S4 in the Supplementary Material).

### 3.1 BBS and WBS

Significantly stronger BBS (Q<0.05, FDR-corrected) during COOP (COOP > IND) was found in many (113) ch-ch-fr, whereas significantly stronger BBS during IND (COOP < IND) was limited (9). The frequency band with the smallest p-value was selected as the representative ch-ch-fr for a given channel-pair. Consequently, there were 24 representative ch-ch-fr, among which 21 showed significantly stronger BBS during COOP (5 were from identical-channel-pairs, 16 were from different-channel-pairs) (Table S1 in the Supplementary Material).

**Fig. 4:**
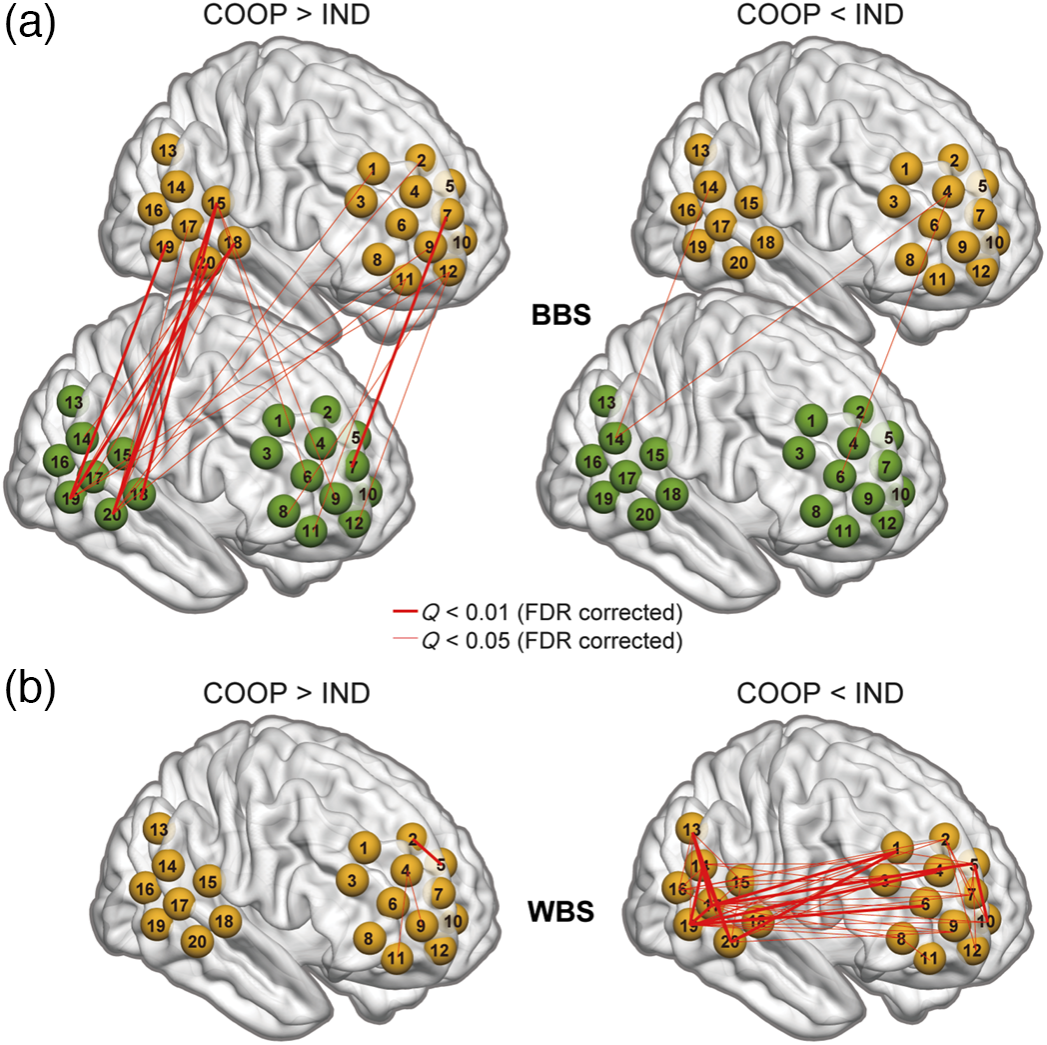
Top significant (Q<0.05, FDR corrected) (a) BBS and (b) WBS obtained from oxy-Hb data. Left panel: COOP > IND; Right panel: COOP < IND. The width of the lines reflects the Q value (the smaller the Q value is, the thicker the line is). Brain images were created using BrainNetViewer.^26^

The positions and connections of the significant between-brain channel-pairs are shown in Fig. 4(a). Significantly stronger BBS (COOP > IND, left) occurred not only between two participants’ temporal regions or the prefrontal cortex (PFC), but also between the temporal region of one participant and the PFC of the other participant. In particular, brain activity in CH15 (four connections), CH18 (five connections), CH19 (five connections), and CH20 (eight connections) showed the most robust synchronization with the brain activity of the partner. According to virtual spatial registration,^28^ CH15 [right superior temporal gyrus (rSTG) 66%] and CH18 [rSTG 78%, right middle temporal gyrus (rMTG) 22%] approximately represented the rSTG region, and CH19 (rMTG 73%) and CH20 (rMTG 100%) approximately represented the rMTG region. Very few BBS were found to be significantly stronger during IND (COOP < IND, right).

A representative selection of ch-ch-fr for the WBS, using the same method as BBS, yielded 52 representative ch-ch-fr showing significant differences (Q<0.05) between COOP and IND (Table S2 in the Supplementary Material). As shown in Fig. 4(b), most (49) of these channel-pairs showed significantly stronger WBS during IND (COOP < IND, right). Similar to BBS, other than the WBS within the temporal or PFC region, relatively long-range WBS were also observed between the temporal and PFC areas. Representatively, CH19 (14 connections) and CH20 (seven connections) covering the rMTG and CH15 (four connections) and CH18 (two connections) covering the rSTG function as the central nodes of several significant WBS. Importantly, during COOP, a single brain shows many more connections to the partner’s brain [Fig. 4(a), left], in contrast to very limited connectivity within itself [Fig. 4(b), left].

**Fig. 5:**
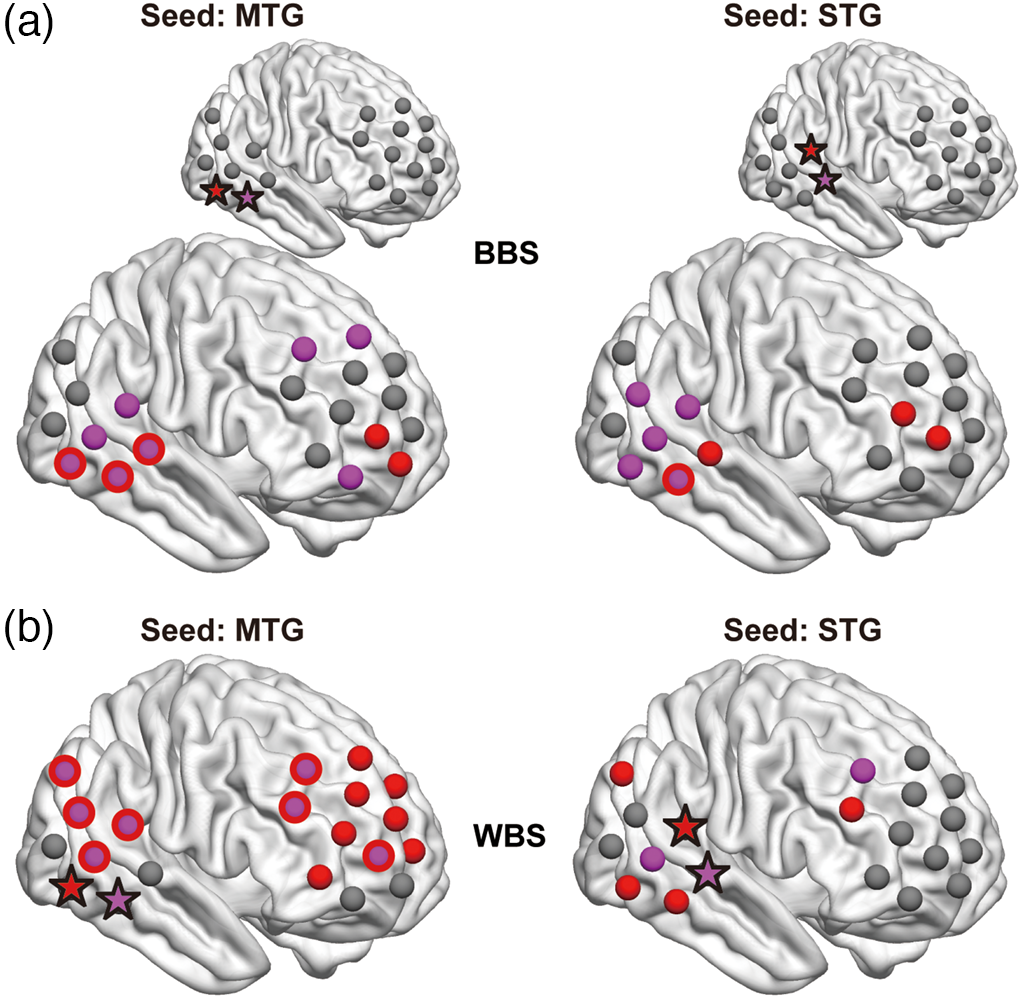
Connectivity map using MTG (left panel) and STG (right panel) as seeds for BBS [(a) COOP > IND] and WBS [(b) COOP < IND] obtained from oxy-Hb data. The two stars (red and purple) represent the two channels that serve as seeds (MTG or STG). The red dots represent channels that synchronized with the red star channel, and the purple dots represent channels that synchronized with the purple star channel. The purple dots with red frames represent channels that synchronized with both red and purple star channels. Brain images were created using BrainNetViewer.^26^

Figure 5 shows the representative significantly stronger BBS (COOP > IND) and WBS (COOP < IND) using rMTG (left panel) and rSTG (right panel) as seeds. Brain activity in a similar network, including the rMTG, rSTG, right frontopolar (rFP), right dorsomedial PFC (rDMPFC), and right dorsolateral PFC (rDLPFC), can be observed under both conditions. It seems that this network functions within one brain during IND, whereas the two brains build this network together during COOP.

Significant synchronizations shown in Fig. 4 did not show significant correlation with the results of the questionnaire on the task (all Q>0.05, Sec. 1.8 in the Supplementary Material).

### 3.2 WTC-GLM

**Fig. 6:**
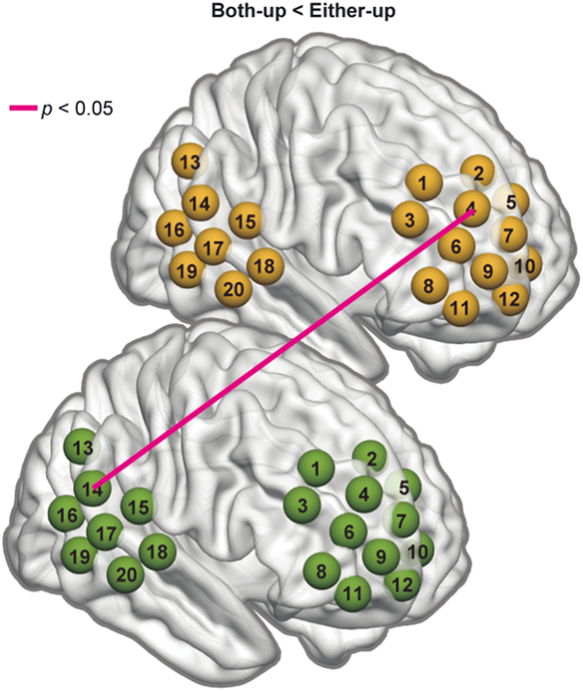
Results of the WTC-GLM analysis (N=23 dyads) using oxy-Hb data. Synchronized activity between the two brains (CH4 and CH14, representing the rDLPFC and the rTPJ, respectively) was significantly more related to the either-up regressor than the both-up regressor. The result was the same for deoxy-Hb data. Brain images were created using BrainNetViewer.^26^

The results above for BBS of oxy-Hb revealed significantly stronger synchronization in COOP than in IND for many channel-pairs. The GLM analysis further elucidated how these synchronizations were specifically related to particular social behaviors such as face-up events. Comparison of β values between the regressors both-up and either-up obtained from the GLM analysis revealed a significant difference in one ch-ch-fr, CH4 (rDLPFC 90%) and CH14 [right temporo-parietal junction (rTPJ): posterior STG 70.6%, SMG 21.8%] at 0.09 Hz [t=−3.21, p=0.0025 (p<0.05/15), Bonferroni’s correction] (Fig. 6). In other words, the synchronization of brain activity in CH4 of one participant and CH14 of the partner was more related to either-up events than both-up events. It is worth noting that the final dataset for this channel-pair came from 23 dyads with valid data for both WTC and behavior analyses. Additionally, we obtained the same result for BBS of deoxy-Hb [t=−3.21, p=0.0025 (p<0.05/15), Bonferroni’s correction].

## 4 Discussion

This study used fNIRS hyperscanning to investigate the neural correlates of cooperative creation during an unrestricted turn-based computer game with changeable goals. We proposed an approach to extract particular neuronal synchronization related to specific social communicative signals among various behaviors elicited in such a goal-free task. Specifically, we used an automatic behavioral detection technique to mark epochs with targeted social behaviors (face-up) and then examined the relationship between face-up behaviors and neuronal synchronization using a GLM. WTC analysis revealed distinct patterns of brain synchrony in different situations: joint play induced greater BBS among the rSTG, rMTG, rFP, and rDMPFC/rDLPFC across the two brains, whereas independent play induced stronger WBS among similar regions within a single brain. WTC-GLM analysis showed that BBS between the rDLPFC and rTPJ of two brains was significantly more related to the behavior when only one participant raised his/her face than when both members raised their faces.

### 4.1 Two-in-one System

Conventional WTC analysis revealed that joint play elicited significantly stronger BBS in many channel-pairs [Fig. 4(a), left], in contrast with very limited number of BBS that was significantly stronger during independent play [Fig. 4(a), right]. Conversely, independent play induced significantly stronger synchronization within a single brain (WBS) [Fig. 4(b), right]. Interestingly, the localization of significant channels under the two conditions (BBS in COOP and WBS in IND) overlapped to a large degree (Fig. 5). For example, during IND, the brain activity of rMTG (CH19 and CH20) synchronized with that of rSTG, rDMPFC/rDLPFC, and rFP within a single brain [Fig. 5(b), left], whereas during COOP, the activity of the rMTG in one brain synchronized with that of the rSTG, rDLPFC, and rFP in the other brain [Fig. 5(a), left]. These findings indicate an intriguing phenomenon. Neuron populations responsive to this creative task within one brain activated simultaneously with similar neuron populations within the other brain when the dyad cooperate to complete the task, as if the two brains function together as a single system for creative problem solving. Importantly, in COOP, WBS was largely inhibited [Fig. 4(b), left] compared with BBS [Fig. 4(a), left], probably because one must allocate more cognitive load to adjust to the partner. Another possibility would be that the reduced WBS may indicate redistribution of energy/blood flow toward the language area. However, our results did not show such an effect in the Wernicke’s area, and our probe did not cover the Broca’s area properly, so we could not test this possibility. These phenomena are consistent with the notion of “we-mode,” in which interacting agents share their minds in a collective mode.^35^ This mode has been described as facilitating interaction by accelerating access to other’s cognition.^35^^,^^36^ Our participants may have achieved their shared goal (to create a room that satisfies both parties) using such a system. It is interesting for future study to also examine whether we-mode is still there when dyads are involved in negative social interactions such as to hinder the partner.

### 4.2 Possible Cognitive Functions Underlying Synchronized Brain Areas

What specific cognitive processes do the BBS between MTG/STG and FP/DLPFC reflect during COOP? Several recent single-brain studies have reported the involvement of the MTG region in creativity-related tasks.^37^^,^^38^ The STG region has also been found to contribute to the neural process of ideation, such as realizing solutions during creative problem solving.^37^^,^^39^^,^^40^ Although the MTG and STG regions usually respond to auditory input, they did not show vocalization-related synchronization according to our WTC-GLM analysis (Sec. 2.2 and Fig. S6 in the Supplementary Material). Therefore, our results on BBS in the MTG/STG regions were not directly related to speech but may be neural correlates of the cognitive process for creative tasks.

Although rarely reported as neural-coupling in the PFC in previous studies, there has been an increase in recent reports of synchronization in the FP^14^^,^^41^^–^^43^ and the DLPFC.^12^^,^^14^^,^^42^^,^^43^ These areas are crucial in neural processes of not only attention and executive decision but also theory of mind,^41^^,^^44^^,^^45^ all of which are essential elements for successful communication. The DLPFC is a versatile brain region that contributes to various neural processes, except for its classic functions. We further discuss its possible functioning within our context in the following section, combined with our WTC-GLM results.

Most previous fNIRS hyperscanning studies focused on brain synchronization in the same channels of dyads. Recent research also discovered synchrony between different channels/regions.^10^^,^^12^ Our findings on synchronization between the PFC region of one person and the temporal region of the other person provided further evidence for relatively long-range cross-brain synchrony during social interactions. Cañigueral et al.^12^ also found synchronization between the DLPFC of one brain and the rTPJ of the other when participants were involved in an implicit interactive task demanding mentalizing, anticipation, and strategic decisions over each other’s choices. Because the hub-like CH15 corresponds to the rTPJ, our study again indicated a significant role for rTPJ during interactions. We also discuss the function of rTPJ in the following section in relation to the WTC-GLM results.

In summary, the significantly stronger BBS in the FP/DLPFC of two brains and that projecting between the FP/DLPFC of one brain and the MTG/STG of the other brain may be neural indicators for (1) endeavors for creative problem solving of the dyads toward an abstract shared goal (to create a room that satisfies both parties) and (2) information exchange during such cooperation that requires constantly attending to and reading others’ intentions.

### 4.3 Further Insights via WTC-GLM Analysis

As we adopted a task allowing nearly unconstrained interaction, various social behaviors would have been associated with the large number of synchronized channels obtained using conventional WTC analysis. To clarify the specific relationships between certain types of interactive behavior and certain types of brain synchrony, we applied GLM to the WTC results (WTC-GLM) in an event-related manner, wherein the events were social behaviors extracted automatically using computer vision. We chose face-up events as the target behavior because it should approximately correspond to eye gaze, reflecting a person’s thoughts or intentions.^11^ We assumed that concurrent face-up reflects mutual gaze and single participant’s face-up corresponds to a form of communicative intention. This includes talking to the partner, observing the partner’s behavior, and/or guessing the partner’s intention.

A comparison of the WTC-GLM results between the both-up and either-up regressors extracted one significant between-brain channel-pair (CH4 and CH14 corresponding to rDLPFC and rTPJ) with a synchronization that was more related to the either-up regressor (Fig. 6). To evaluate any influence from head-motion artifacts, we also analyzed the relationship between face-up motion synchronization and regressors (Sec. 2.1 in the Supplementary Material). No significant correlation was found, so our WTC-GLM results were unlikely to be related to the motion synchronization of face-up behaviors. We suppose that this BBS is related to the mentalization process. TPJ is a broad region inclusive of SMG, AG, and posterior STG (pSTG), which serves as a crucial part of the MENT^46^^,^^47^ and is considered central in representing mental states during the theory of mind tasks.^48^^,^^49^ Regardless of the measurement modality, several hyperscanning studies have reported increased synchrony in TPJ between individuals engaged in social interactive tasks.^9^^,^^14^^,^^21^^,^^23^ Using fMRI hyperscanning, Abe et al.^23^ confirmed inter-brain synchronization in both the anterior and posterior parts of rTPJ and suggested the anterior rTPJ’s role in directing attention to the partner’s behavior and the posterior rTPJ’s role in coordinating self and other behaviors. As to the DLPFC, in addition to its generally accepted functions such as attention and goal maintenance,^50^ it has also been reported to play key roles in monitoring responses and inhibiting self-centered behavior and ideas.^50^^–^^52^ Using fNIRS hyperscanning, Xue et al.^14^ found significant synchronization of the rDLPFC in their creativity-demanding tasks, especially for dyads who were more willing to cooperate and therefore possibly more inclined to prioritize the partner’s idea.

**Fig. 7:**
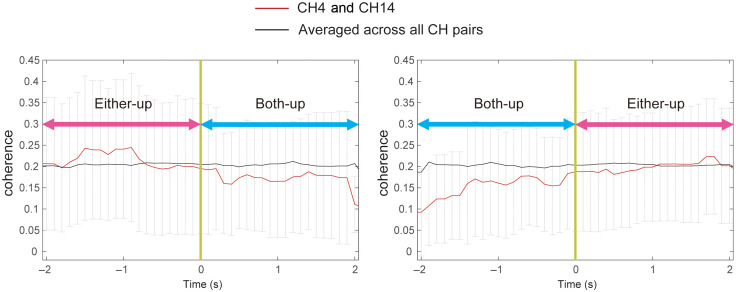
Time series of the WTC obtained from oxy-Hb between CH4 (rDLPFC) of one brain and CH 14 (rTPJ) of the other brain at 0.09 Hz, which were averaged from those dyads who had either-up and both-up events. The “0” point represents the starting point of the both-up event (left panel) and the ending point of the both-up event (right panel), respectively. The black line is the averaged WTC of all channel combinations at 0.09 Hz (baseline level). The red line is WTC between CH4 and CH14 of the two brains.

In light of this evidence, one possible explanation for the stronger relationship between the BBS of rTPJ and rDLPFC and the either-up regressor is as follows. When both members raised their faces (both-up), they were better able to understand each other’s intentions and feelings via facial information, whereas facial information exchange is largely limited to one direction during either-up so that more effort is required to infer the partner’s (say, participant B) intention by observing facial information (functioning of the rTPJ) and adjust one’s (participant A) own mind and behavior accordingly (functioning of the rTPJ). Meanwhile, participant B noticed/attended to this gaze (functioning of the rDLPFC), understood participant A’s thought, and adjusted his/her own idea and behavior (functioning of the rDLPFC) to design a room that also satisfied participant A. Further analysis partly supports this assumption. The WTC time series between the rDLPFC and rTPJ was aligned with the timing of both-up events (the “0” point) (Fig. 7). It showed that synchronization was stronger during the several seconds immediately before the both-up period started but declined during the both-up period (left panel). Similarly, synchronization was generally weaker during the both-up event, but gradually increased after the both-up period ended (right panel). Because the afore-mentioned synchronizations were significantly more pronounced when only one participant raised his/her head, it is not likely that such synchronization was due to concurrent head movements. Furthermore, we also obtained the same results for deoxy-Hb signals, which are rather robust to physiological noises.

Interestingly, the synchronization between the rTPJ (CH14) of the two brains and between the rTPJ of one brain and the rDLPFC (CH4) of the other brain was significantly stronger during independent play [Fig. 4(a), right]. This finding may also reflect the functioning of the network when the two persons could not see their partner’s face or body and had to wait until the partner completed their turn to start their own turn. This process may indicate mentalization of time and turn-taking, which was not as necessary for joint play.

Additionally, the occurrence rate of both-up and either-up events in the four types of behavior logs (item selection, item placement, ok, and cancel) during COOP showed no significant difference (Kruskal–Wallis’s test, p<0.05; Fig. S4 in the Supplementary Material). This result excludes the possibility that the specific neural synchronization related to the face-up regressor is biased toward a certain game behavior. Rather, the observed relationship between social behavior and neural synchronization is universal in the present naturalistic cooperative setting.

### 4.4 Significance of Our Proposed Analytical Approach and Limitation

Neurocognitive research focusing on real dynamic interactions using hyperscanning techniques is an emerging field that has provided many valuable insights into understanding the social brain.^1^^,^^2^^,^^4^ Recently, naturalistic stimuli in more ecologically valid situations have provided novel evidence for social neuroscience. Such rich behavioral information from naturalistic stimuli can contribute to a more comprehensive understanding of the social brain. Meanwhile, conventional methods used to examine brain–behavior relationships (such as manual encoding of social signals from videotapes by multiple trained coders) will be limited as big data emerges from social neuroscience in naturalistic settings. The trend toward big data underscores the importance of developing new analytical techniques that can reveal brain–behavior relationships more efficiently.

This study sought to address this issue using automated computer vision techniques to extract metrics of human behavior during naturalistic social interactions. We focused on face-up events in our cooperative creativity task. Although holding less social information than pitch angle, the roll and yaw angles of the participant’s head should also be considered in future analyses for precision. As the first attempt to apply this automatic method, this study targeted one type of salient human motion. In future analyses, we could apply this method to more detailed social behaviors, such as facial expressions and verbal behaviors. For such attempts, techniques that can effectively videorecord these multidimensional social behaviors are necessary to better understand the neural correlates of dynamic social interactions under ecologically valid conditions.

One could claim that a limitation of our study is a difference in the speech condition between COOP and IND. However, this study aimed to examine natural interaction for creative goal-free task and to explore a method of detecting behavior-specific synchronization in natural states, so we prioritized naturalness by allowing speech during COOP. We do not deny a possibility that stronger BBS during joint play was due to various interactive signals including speech. Regardless, it is interesting that the BBS during COOP showed similar brain connections to those of WBS during IND, which did not include speech. To examine the possible impact of speech, we additionally applied our WTC-GLM paradigm to isolate vocalization-related synchronization from WTC during COOP (Sec. 2.2 in the Supplementary Material). Although many BBS showed a significant relationship with vocalization, there was no overlap of channel-pair between COOP-related BBS and vocalization-related BBS. These results support that the brain synchronizations during COOP do not directly reflect speech factors. Furthermore, we also confirmed that the ratio of vocalization in the either-up periods was not significantly different from that in the both-up or “other” periods (Fig. S7 in the Supplementary Material). This suggests that either-up specific BBS (rTPJ and rDLPFC) was unlikely to relate to speech factors. It is worth noting that our audio-recording quality prevented us from dissociating the identity and content of speech. Future studies with higher quality audio-recording may provide further insights into speech factors.

## 5 Conclusion

Our cooperative creativity task induced robust BBS that could be characterized as functioning in a two-in-one system. This brain-coupling, represented as dynamic interplays of the STG/MTG regions and the FP/DLPFC regions between the two brains, was assumed to reflect cooperative creative problem solving. Notably, WBS was largely restricted during this mode, probably due to the endeavor to adjust one’s mind and behavior to the partner. Furthermore, we uncovered the underlying relationship between certain neural correlates and specific social behaviors among numerous brain synchronizations by combining GLM analysis with automatic behavioral detection. Our proposed analytical approach may provide insights and avenues for future work in interactive social neuroscience.

## Code, Data, and Materials Availability

The datasets generated and/or analyzed during the current study are available from the corresponding author upon reasonable request.
