# Supplementary material for: Two-in-one system and behavior-specific brain synchrony during goal-free cooperative creation: an analytical approach combining automated behavioral classification and the event-related generalized linear model
Source: Neurophotonics. 2023 Feb 10;10(1):013511. doi: 10.1117/1.NPh.10.1.013511 (PMC9917717; doi:10.1117/1.NPh.10.1.013511)
Supplement: Supplementary file 1 [file NPh_010_013511_SD001.docx]

1. **Supplementary Method**
   1. Interior design game

The interior design game is a browser game that runs on a server. Two client computers with a touch panel display (Lenovo Yoga 900, 13.3 inches) were connected to the server with remote desktop software (TeamViewer 11) via LAN cables to play the game in turn by the two participants in a dyad through the two client computers. The operation of the game on one client computer was projected almost simultaneously on the screen of the other client computer, with a delay of less than the NIRS sampling period (100 ms).

- 1. Details of videotaping

The entire procedure of the experiment was videotaped using two video cameras (Panasonic, HC-V360M, Full HD, 29.97 fps). Each camera mainly focused on one participant in a dyad. To synchronize the video recording with the fNIRS signals, both cameras also recorded the ongoing procedure of the game projected on one large display, which was identical to the dyad’s touch panel displays in real-time. This large display was positioned on the side of the dyad to be out of the participants’ sight (see Fig.1. I), II), the lower part of the right panel).

- 1. Details of the probe set

For each participant, two 3 × 3 probe pads were used. The positions of the probes were determined according to the international 10-20 system. One 3 × 3 probe pad was placed on the right prefrontal region (five emitters and four detectors, 30 mm probe distance, forming 12 measurement channels (CH)). The bottom line of the probe was placed along with the Fp1–Fp2 line, with the corner detector positioned at Fpz (Fig.1. III), left panel). The other 3 × 3 probe pad (partially used, four emitters, and three detectors, forming eight channels) was placed on the right temporal region. The bottom line of the probe was placed along the T4-F8 line, with CH20 positioned at T4 (Fig.1. III), right panel).

- 1. Details of WTC analysis

As each participant in the dyad was measured in 20 channels, there were 20 identical channel pairs and 190 different channel pairs (e.g., CH1–CH20 and CH20–CH1 were considered as one different channel pair for a dyad), totalling 210 channel pairs for each dyad. For each identical channel pair, one WTC value was obtained, whereas for each different channel pair, two WTC values were obtained (e.g., WTC between CH1 of participant A and CH20 of participant B, and WTC between CH20 of participant A and CH1 of participant B).

- 1. Selection of frequency bands and details of correction for multiple comparisons

Since we aimed to examine neural synchrony elicited by spontaneous social interactions that are highly unpredictable, it may be not sufficient to only target narrow frequency range when analyzing the brain synchrony related to social interactions. Instead, we first used a wide frequency range (0.0072−3.68 Hz) and adopted 91 frequency bands according to the rationale of the original WTC method at 1/10 octave intervals (Grinsted, et al., 2004), and then we calculated the coherence of all possible between-brain and within-brain channel-pairs at each frequency band. Specifically, the time-frequency resolution of WTC depends on the shape of the mother wavelet and the number of scales per octave. For the commonly used mother wavelet (i.e., Morlet wavelet), the optimal number of scales per octave is suggested to be more than 10 (Grinsted et al., 2004).

This would lead to a large number of ch-ch-fr combinations. To minimize the possibility of type I error as much as possible, we used Storey’s false discovery rate (FDR) for correction of multiple comparisons. Since FDR utilizes the distribution of *p*-values to obtain the *Q*-value, practically, all *p*-values obtained (using the wider frequency range of 0.0072−3.68 Hz) are needed for this step. For FDR correction, we treated the *Q*-values from identical channel pairs and different channel pairs identically, although they were obtained from different sample sizes (i.e., 39 samples for each identical channel pair and 78 samples for each different channel pair). Then, for the WTC analysis (BBS & WBS), we focused on the significant channel-pairs at a frequency range of 0.03−0.1 Hz, which is usually referred to as neurogenic activation [see ref 1 & 2]. Our WTC-GLM analysis also targeted this frequency range (0.03−0.1Hz).

Reference

[1] Stefanovska, A. (2007). Coupled oscillatros: complex but not complicated cardiovascular and brain interactions. IEEE Eng. Med. Biol. Mag. 26, 25–29. doi: 10.1109/emb.2007.907088

[2] Zhang, X., Noah, J. A., Dravida, S., and Hirsch, J. (2020). Optimization of wavelet coherence analysis as a measure of neural synchrony during hyperscanning using functional near-infrared spectroscopy. Neurophotonics 7:015010. doi: 10.1117/1.NPh.7.1.015010

- 1. Details of face orientation analysis

We extracted the face orientation data using automatic tracking in OpenFace 2.1.0. Periods with confidence values for landmark detection smaller than 0.8 were considered unreliable periods. Unreliable periods and 30 frames (approximately 1 s) of pre- and post-unreliable periods were excluded from the face orientation data.

As the display of touch panel of each participant was placed on a desk, the participants had to raise their face to look at the partner. We confirmed from the video that the participants raised their faces when they actively engaged in social communication with their partner, which was chiefly reflected in the change in pitch angle.

- 1. Details of WTC-GLM analysis

For the GLM analysis, we used the WTC without pre-whitening because it distorts the WTC’s temporal structure. Since WTC is temporal-spectral synchrony between fNIRS signals, rather than hemodynamic data, we modelled the active epochs in the GLM regressors using box-car shapes.

Among the 39 dyads, only those with valid epochs that satisfied the following criteria were used in the GLM analysis: both participants of a dyad had 1) reliable face orientation data when the pitch angle of each participant’s face orientation was above 2 SD of their own data (Fig.3); and 2) usable fNIRS data for WTC calculation. Note that because the number of dyads with usable fNIRS data differs according to ch-ch-fr, the number of dyads that satisfy both criteria also vary.

- 1. Details of Spearman’s correlation analysis

For Spearman’s correlation analysis between condition-induced significant WTC and subjective factors (scores of AQ and task questionnaire), the mean WTC of the two cooperative play (COOP) sessions for a particular ch-ch-fr was selected as the dependent variable. The product of the two scores for each dyad was used for the task questionnaire scores.


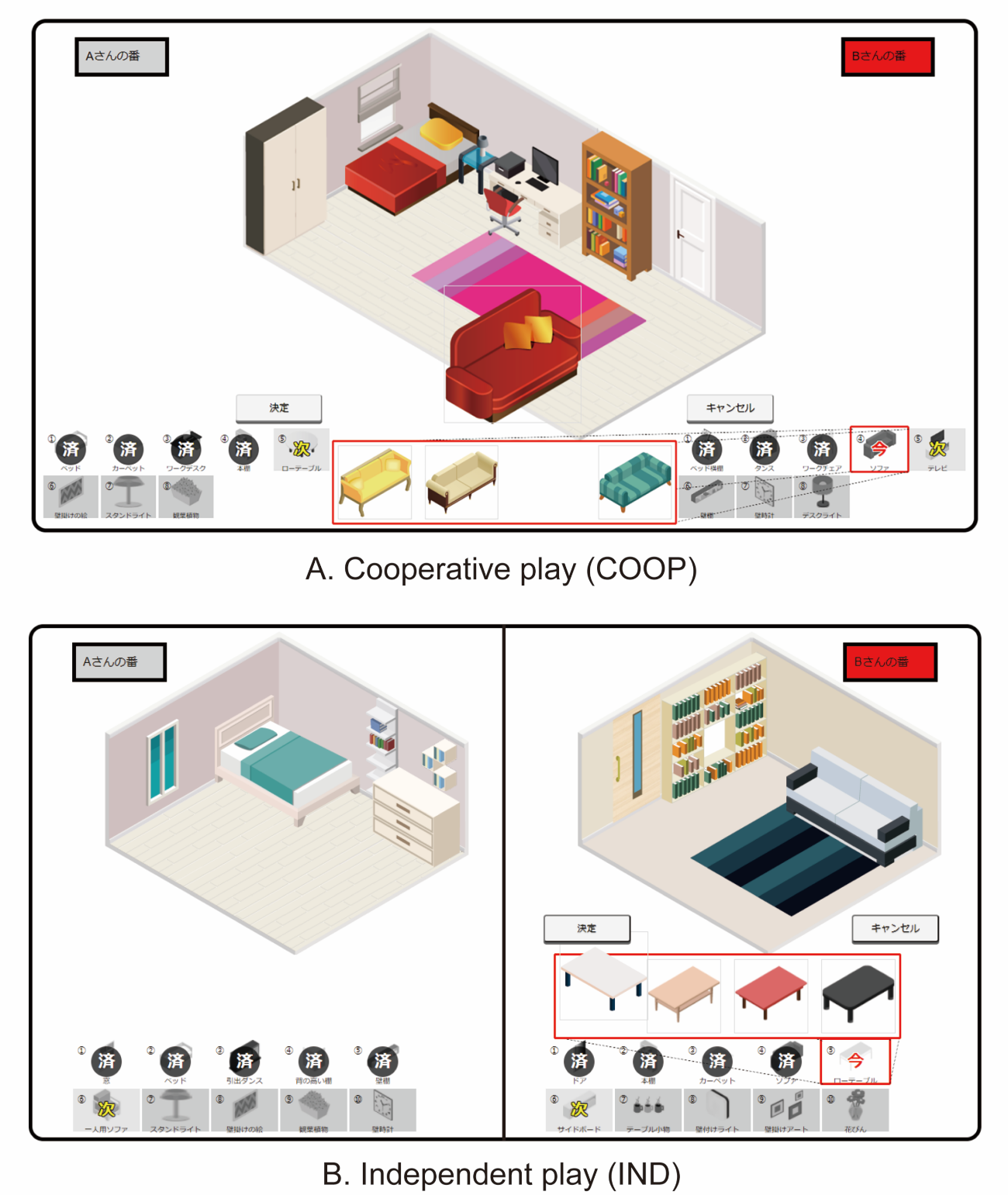


Supplementary Figure 1. The midway layout of the furnished room in one cooperative (COOP) session (upper panel) and one independent (IND) session (lower panel) from the sample dyad that is shown in Figure 1 (in the manuscript). The game is in a turn-based manner. For each turn, four candidates for a specific piece of furniture or interior (e.g., a bed or lamp) were presented, and the on-duty participant was asked to choose one from the four and place it in a position that suits the room well. The selection and placement of a furniture/interior could be modified (press the “cancel” button) as much as necessary until a decision was made (press the “OK” button). After pressing the “OK” button, no modification was allowed, and the next turn for the other person started. For the COOP sessions (upper panel), there were 16 types of furniture/interiors (16 turns) in total, and the sequence of their appearance was fixed. The dyad had to complete the design of the present interior to proceed to the next interior. When the design of the 16 items was completed, “Mission accomplished” (in Japanese) was presented on the screen to intensify their feeling of accomplishment. The whole landscape of the to-be-designed room was presented on the screen of each participant (see Figure 1 in the manuscript). Thus, they shared the same view of the ongoing game, including the manipulations (e.g., selection and placement of items) by their partners. For the IND sessions (lower panel), the general rules and procedures of the game were identical to those of COOP, except that the total number of items was 10 for each participant’s room. Furthermore, since a partition board physically separated them, they could not see their partner and were never able to see their partner’s room with their touch panel (half of the screen showing the landscape of their partner’s room was hidden with a piece of hard board aligned with the black vertical line). To control the waiting time to be similar to that in the COOP sessions, each participant had to wait for their own turn until the partner finished, which was indicated on the screen of their touch panel (upper left corner for participant A and upper right corner for participant B). When the indicator changed from grey colour to red colour, the on-duty participant could start their turn.


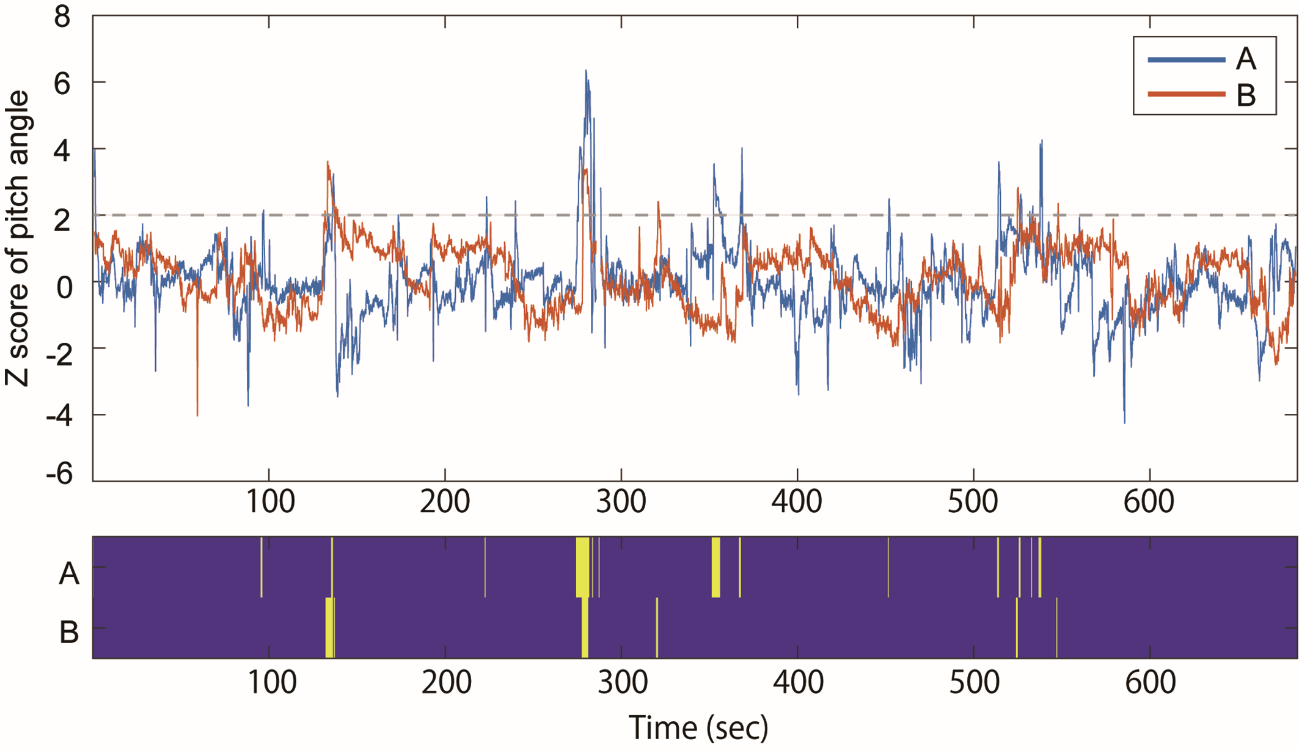


Supplementary Figure 2. Upper panel: the *z*-score-transformed pitch angles of face orientations from a sample dyad (A and B, respectively). The 2 SD threshold is shown as the dotted line. Lower panel: the automatically detected face-up events from this sample dyad are shown as yellow bars.


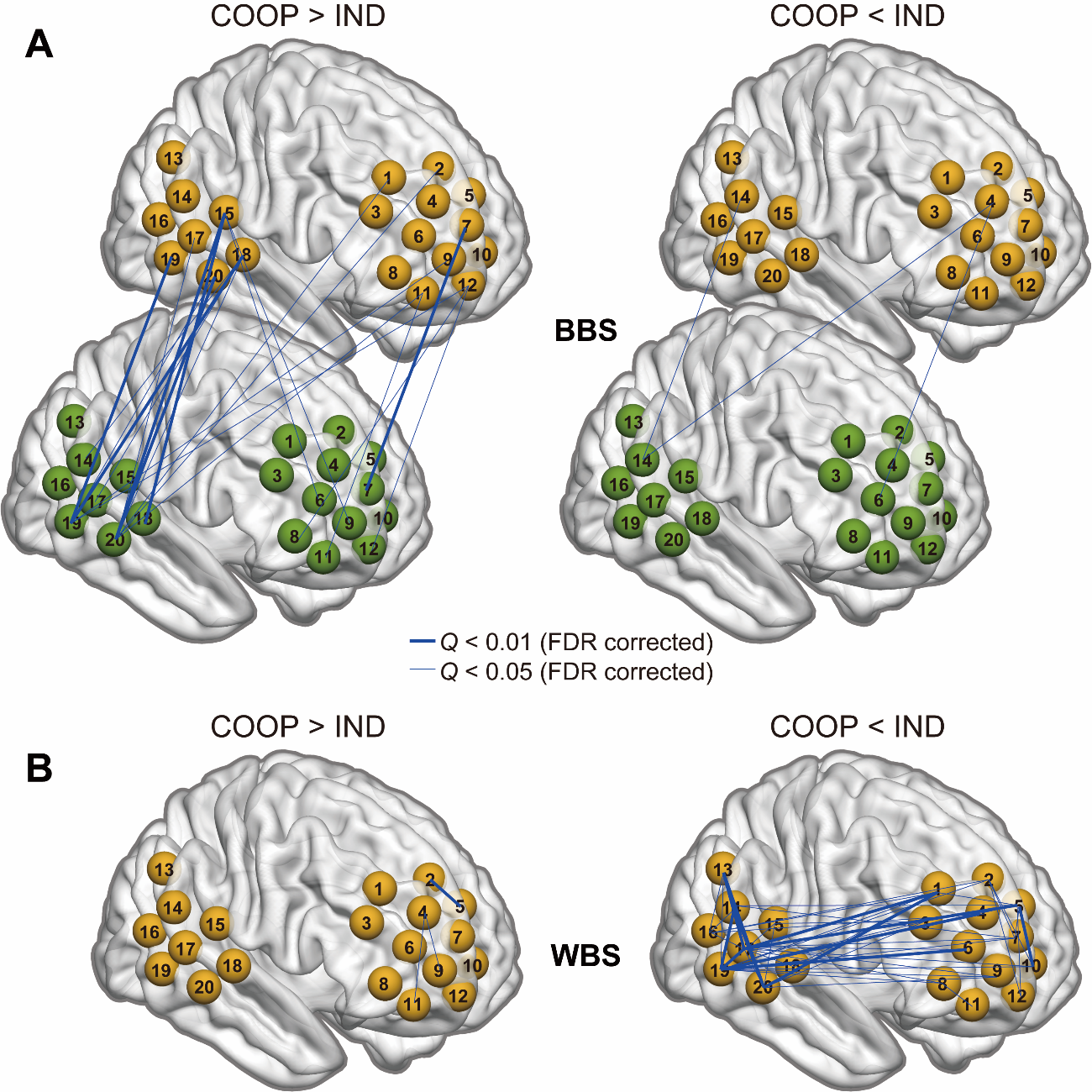


Supplementary Figure 3. Top significant (Q < 0.05, FDR corrected) between-brain synchronization (BBS) (panel A) and within-brain synchronization (WBS) (panel B) obtained from deoxy-Hb data. Left panel: COOP > IND; Right panel: COOP < IND. The width of the lines reflects the *Q* value (the smaller the Q value, the thicker the line). Brain images were created using BrainNetViewer (http://www.nitrc.org/projects/bnv) (Xia et al., 2013).


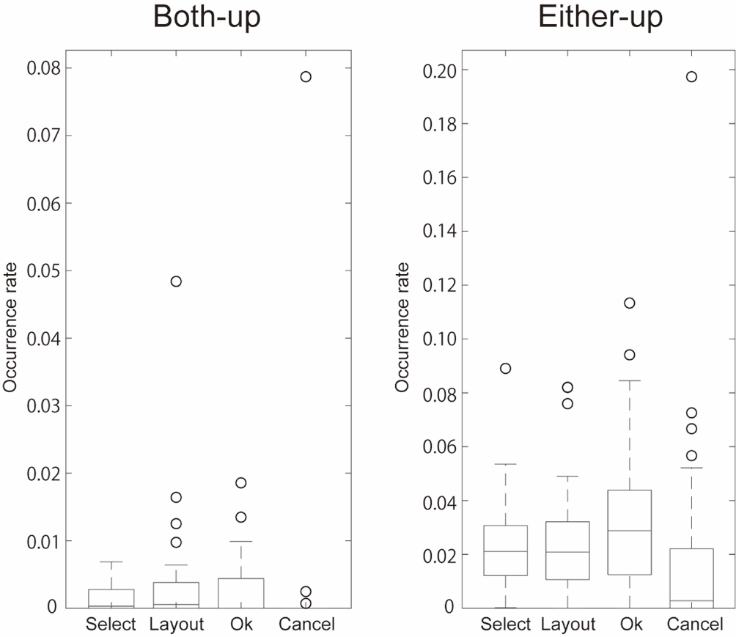


Supplementary Figure 4. Box plot showing the occurrence rate of face-up events during the four types of game logs during the cooperative (COOP) sessions. There were no significant differences in the occurrence rate of face-up (“both-up” and “either-up”) events between the game logs (Kruskal–Wallis’s test, *p* < 0.05). Select: item selection; Layout: item placement; OK: item decision; and Cancel: redo from the start. Left panel: “both-up” events. Right panel: “either-up” events.

1. **Supplementary analysis & results**

2.1 The influence of the "face-up" motion synchronization on the WTC-GLM results

To confirm whether synchronized "face-up" motion-artifacts would have an impact on our WTC-GLM results or not, we calculated the correlation between our regressors (“both-up” and “either-up”) and the "face-up" motion synchronization. The amount of "face-up" motion synchronization was obtained by computing the WTC between the dyad’s head motion vectors (i.e., pitch angles, abbreviated as PA-WTC). Then, we calculated the Spearman correlation between our regressors and the PA-WTC for each frequency band. The parameters of the PA-WTC were the same as the WTC analysis for fNIRS data. There was no significant correlation between them (Supplementary Figure [SFig.] 5). This result suggests that our WTC-GLM results were unlikely to be related to the "face-up" motion synchronization.


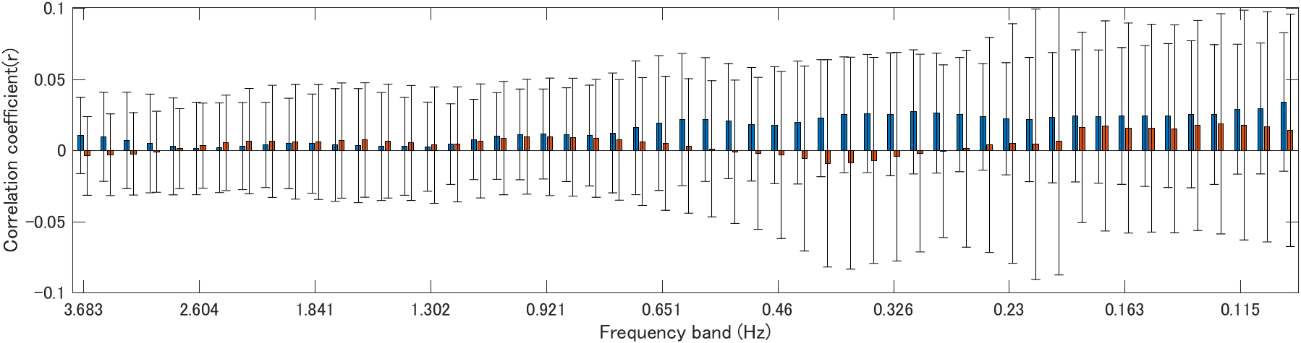


Supplementary Figure 5. Correlation coefficient between the PA-WTC and WTC-GLM regressors (blue bars: "both-up”, red bars: “either-up”) at each frequency band. The horizontal axis indicates the frequency band of PA-WTC. Each bar and error bar represent the mean and the standard deviation of correlation coefficient, respectively.

2.2 The influence of vocal exchanges on the WTC-GLM results

Additional analyses were performed to confirm the effect of voice exchange on our main results. Since the audio-recording quality of the present study prevented us from identifying the speakers and speaking contents, we only focused on the events of voice exchanges in the COOP condition. The presence of vocalizations was estimated for each audio file by using the Voice-Activity-Detection (VAD), a pre-trained Bidirectional Long Short-Term Memory network (‘*speechDetectNet*’) and a configured ‘*audioFeatureExtractor*’ object, which are provided in Matlab 2021a: <https://www.mathworks.com/help/deeplearning/ug/voice-activity-detection-in-noise-using-deep-learning.html>

The time series of the estimated binary vector (i.e., 1 for “voice”, and 0 for “no-voice”) were down-sampled to 10 Hz. To obtain robust estimation results of vocalization periods, we only adopted the matched period of both vectors from the two video cameras. We conducted a WTC-GLM analysis using the “voice” and “no-voice” binary vectors as regressors on the WTC in COOP. The WTC-GLM analysis pipeline was identical to that performed using face-up events as regressors in the main text of manuscript. The βs for the vocalization regressors in each ch-ch-fr were compared with the baseline (i.e., zeros) using paired *t*-tests. FDR-correction was utilized for multiple comparisons. The WTC of one representative frequency band (which showed the smallest *p*-value) was chosen for each channel-pair. The significant channel-pairs related to vocalization were shown in SFig.6B. For comparison, channel-pairs showing significantly stronger synchronization during COOP (COOP > IND, BBS) were shown in SFig.6A (the same with Fig.4A in the manuscript). There was no overlap of channel-pair between the two situations, which indicates that our WTC results were unlikely to be related to voice exchanges.


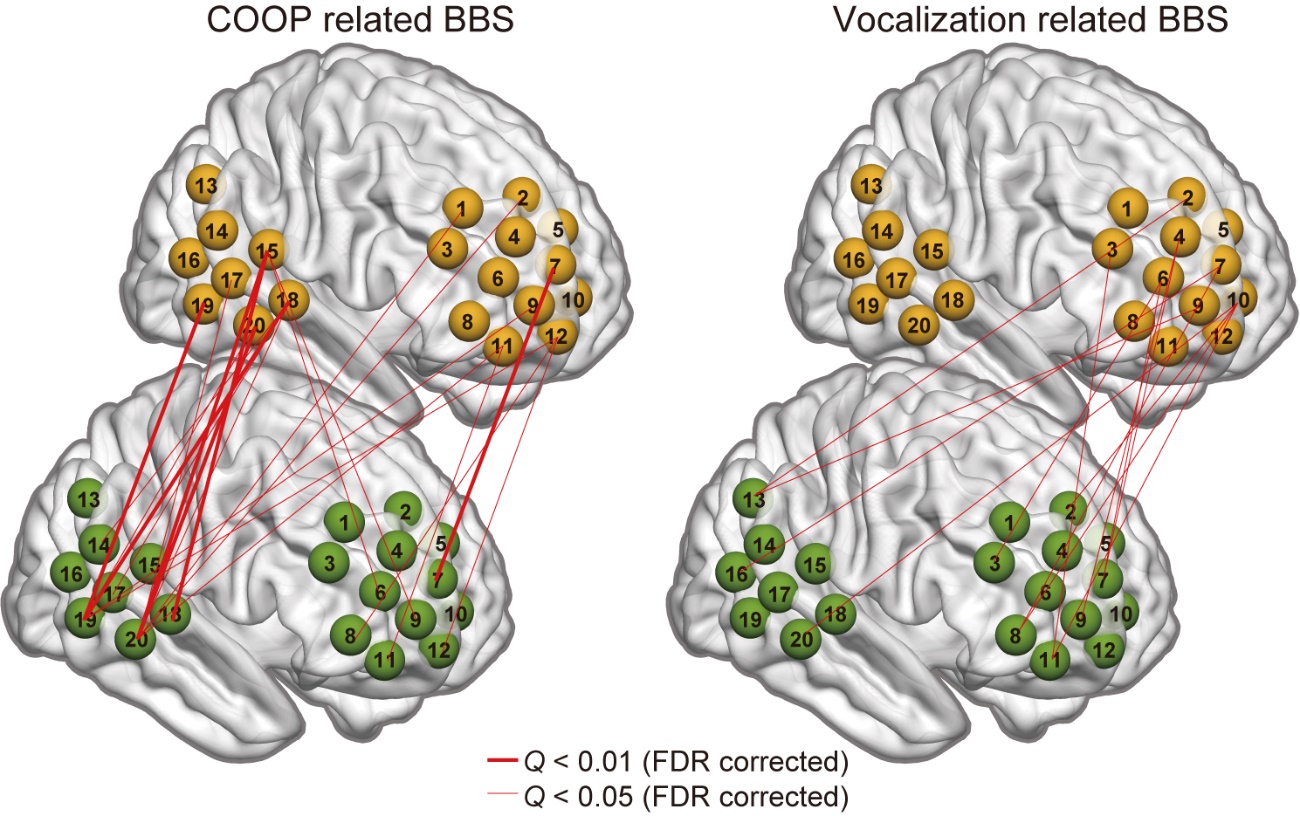


Supplementary Figure 6. A comparison of COOP related BBS (left) and vocalization related BBS (right). No overlap of channel-pair was found.

In addition, we investigated the overlap period of detected vocalization with each of our regressors ("both-up", "either-up", and “other”) in the WTC-GLM analysis. The speech ratio (duration of vocalization/total duration) for the regressors were compared (Friedman’s test, n = 24, χ2 = 13.08, *p* < 0.01). Post-hoc comparisons revealed no significant difference (*p* > 0.05, Bonferroni’s correction) between the “either-up” periods and the “both-up” periods or between the “either-up” periods and the “other” periods (SFig.7). While we found a significantly larger beta value in our WTC-GLM for the “either-up” periods, we consider that vocal exchanges would not unlikely to have influenced our WTC-GLM results.


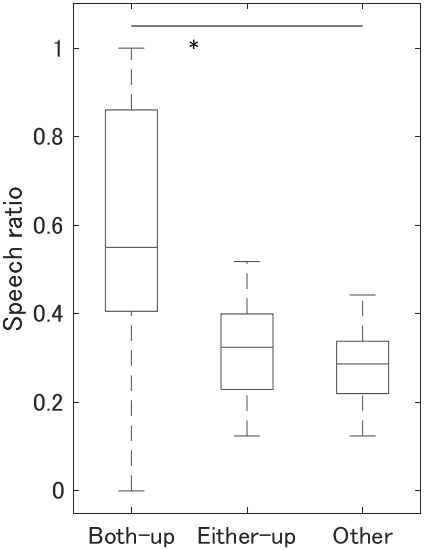


Supplementary Figure 7. Detected speech ratio in the periods of each regressor (“both-up”, “either-up”, and “other”) used in the WTC-GLM. Boxplot shows the speech ratio (duration of vocalization/total duration) for each regressor. There was only a significant difference between the “both-up” and “other” periods (*p* < 0.05, Bonferroni’s correction).

Table 1. Significant between-brain synchronisation (BBS) obtained from oxy-Hb data for cooperative play (COOP) > independent play (IND) (left part), and for COOP < IND (right part). FDR-corrected (*Q* < 0.05).

| COOP > IND | | | |  | COOP < IND | | | |
| --- | --- | --- | --- | --- | --- | --- | --- | --- |
| *CHA* | *CHB* | *Freq (Hz)* | *Q value* |  | *CHA* | *CHB* | *Freq (Hz)* | *Q value* |
| 20 | 20 | 0.0661 | 0.0035 |  | 6 | 4 | 0.0935 | 0.0132 |
| 19 | 18 | 0.0501 | 0.0039 |  | 14 | 4 | 0.0935 | 0.0193 |
| 7 | 7 | 0.0380 | 0.0040 |  | 14 | 14 | 0.0872 | 0.0197 |
| 19 | 19 | 0.0814 | 0.0041 |  |  |  |  |  |
| 18 | 15 | 0.0708 | 0.0058 |  |  |  |  |  |
| 20 | 15 | 0.0759 | 0.0059 |  |  |  |  |  |
| 11 | 11 | 0.0661 | 0.0111 |  |  |  |  |  |
| 12 | 12 | 0.0814 | 0.0130 |  |  |  |  |  |
| 20 | 19 | 0.0501 | 0.0183 |  |  |  |  |  |
| 18 | 17 | 0.0537 | 0.0196 |  |  |  |  |  |
| 20 | 17 | 0.0708 | 0.0243 |  |  |  |  |  |
| 15 | 9 | 0.0708 | 0.0273 |  |  |  |  |  |
| 15 | 6 | 0.0501 | 0.0304 |  |  |  |  |  |
| 20 | 2 | 0.0537 | 0.0323 |  |  |  |  |  |
| 20 | 18 | 0.0537 | 0.0375 |  |  |  |  |  |
| 18 | 14 | 0.0501 | 0.0423 |  |  |  |  |  |
| 12 | 8 | 0.0935 | 0.0428 |  |  |  |  |  |
| 19 | 9 | 0.0708 | 0.0448 |  |  |  |  |  |
| 20 | 1 | 0.0617 | 0.0452 |  |  |  |  |  |
| 20 | 11 | 0.0708 | 0.0485 |  |  |  |  |  |
| 19 | 12 | 0.0617 | 0.0493 |  |  |  |  |  |

Table 2. Significant within-brain synchronisation (WBS) obtained from oxy-Hb for cooperative play (COOP) > independent play (IND) (left part) and for COOP < IND (right part). FDR-corrected (*Q* < 0.05).

| COOP > IND | | | |  | COOP < IND | | | |
| --- | --- | --- | --- | --- | --- | --- | --- | --- |
| *CHA* | *CHB* | *Freq (Hz)* | *Q value* |  | *CHA* | *CHB* | *Freq (Hz)* | *Q value* |
| 5 | 2 | 0.0935 | 0.0077 |  | 19 | 17 | 0.0537 | 0.0012 |
| 11 | 4 | 0.0935 | 0.0248 |  | 17 | 13 | 0.0308 | 0.0031 |
| 9 | 4 | 0.0935 | 0.0249 |  | 19 | 4 | 0.0380 | 0.0031 |
|  |  |  |  |  | 19 | 14 | 0.0436 | 0.0031 |
|  |  |  |  |  | 20 | 1 | 0.0331 | 0.0035 |
|  |  |  |  |  | 19 | 6 | 0.0407 | 0.0049 |
|  |  |  |  |  | 17 | 1 | 0.0308 | 0.0059 |
|  |  |  |  |  | 17 | 5 | 0.0380 | 0.0077 |
|  |  |  |  |  | 20 | 14 | 0.0467 | 0.0077 |
|  |  |  |  |  | 10 | 5 | 0.0617 | 0.0080 |
|  |  |  |  |  | 20 | 13 | 0.0331 | 0.0080 |
|  |  |  |  |  | 17 | 10 | 0.0354 | 0.0104 |
|  |  |  |  |  | 19 | 13 | 0.0407 | 0.0121 |
|  |  |  |  |  | 16 | 14 | 0.0467 | 0.0135 |
|  |  |  |  |  | 19 | 10 | 0.0331 | 0.0142 |
|  |  |  |  |  | 12 | 2 | 0.0407 | 0.0159 |
|  |  |  |  |  | 16 | 13 | 0.0501 | 0.0162 |
|  |  |  |  |  | 12 | 5 | 0.0308 | 0.0165 |
|  |  |  |  |  | 19 | 9 | 0.0354 | 0.0165 |
|  |  |  |  |  | 7 | 5 | 0.0380 | 0.0169 |
|  |  |  |  |  | 20 | 15 | 0.0436 | 0.0170 |
|  |  |  |  |  | 17 | 16 | 0.0501 | 0.0178 |
|  |  |  |  |  | 10 | 2 | 0.0501 | 0.0184 |
|  |  |  |  |  | 20 | 17 | 0.0308 | 0.0226 |
|  |  |  |  |  | 18 | 17 | 0.0308 | 0.0241 |
|  |  |  |  |  | 17 | 8 | 0.0380 | 0.0246 |
|  |  |  |  |  | 20 | 3 | 0.0380 | 0.0249 |
|  |  |  |  |  | 19 | 2 | 0.0407 | 0.0260 |
|  |  |  |  |  | 14 | 7 | 0.0407 | 0.0274 |
|  |  |  |  |  | 16 | 2 | 0.0467 | 0.0290 |
|  |  |  |  |  | 15 | 3 | 0.0308 | 0.0294 |
| Table 2 (continued) | | | | | | | | |
| COOP > IND | | | |  | COOP < IND | | | |
| *CHA* | *CHB* | *Freq (Hz)* | *Q value* |  | *CHA* | *CHB* | *Freq (Hz)* | *Q value* |
|  |  |  |  |  | 17 | 9 | 0.0407 | 0.0304 |
|  |  |  |  |  | 19 | 15 | 0.0380 | 0.0304 |
|  |  |  |  |  | 20 | 9 | 0.0407 | 0.0305 |
|  |  |  |  |  | 14 | 5 | 0.0407 | 0.0313 |
|  |  |  |  |  | 19 | 3 | 0.0380 | 0.0316 |
|  |  |  |  |  | 19 | 7 | 0.0354 | 0.0327 |
|  |  |  |  |  | 17 | 7 | 0.0407 | 0.0353 |
|  |  |  |  |  | 16 | 4 | 0.0308 | 0.0400 |
|  |  |  |  |  | 11 | 8 | 0.0935 | 0.0403 |
|  |  |  |  |  | 18 | 1 | 0.0575 | 0.0412 |
|  |  |  |  |  | 17 | 6 | 0.0407 | 0.0429 |
|  |  |  |  |  | 19 | 8 | 0.0380 | 0.0441 |
|  |  |  |  |  | 5 | 1 | 0.0537 | 0.0469 |
|  |  |  |  |  | 7 | 2 | 0.0501 | 0.0469 |
|  |  |  |  |  | 15 | 13 | 0.0407 | 0.0469 |
|  |  |  |  |  | 16 | 1 | 0.0575 | 0.0471 |
|  |  |  |  |  | 19 | 5 | 0.0661 | 0.0472 |
|  |  |  |  |  | 19 | 1 | 0.0354 | 0.0485 |

Table 3. Significant between-brain synchronisation (BBS) obtained from deoxy-Hb data for cooperative play (COOP) > independent play (IND) (left), and for COOP < IND (right). FDR-corrected (*Q* < 0.05).

| COOP > IND | | | |  | COOP < IND | | | |
| --- | --- | --- | --- | --- | --- | --- | --- | --- |
| *CHA* | *CHB* | *Freq (Hz)* | *Q value* |  | *CHA* | *CHB* | *Freq (Hz)* | *Q value* |
| 20 | 20 | 0.0661 | 0.0035 |  | 6 | 4 | 0.0935 | 0.0136 |
| 19 | 18 | 0.0501 | 0.0041 |  | 14 | 14 | 0.0872 | 0.0182 |
| 7 | 7 | 0.0380 | 0.0042 |  | 14 | 4 | 0.0935 | 0.0195 |
| 19 | 19 | 0.0814 | 0.0050 |  |  |  |  |  |
| 18 | 15 | 0.0708 | 0.0059 |  |  |  |  |  |
| 20 | 15 | 0.0759 | 0.0061 |  |  |  |  |  |
| 11 | 11 | 0.0661 | 0.0123 |  |  |  |  |  |
| 12 | 12 | 0.0814 | 0.0123 |  |  |  |  |  |
| 20 | 19 | 0.0501 | 0.0180 |  |  |  |  |  |
| 18 | 17 | 0.0537 | 0.0200 |  |  |  |  |  |
| 20 | 17 | 0.0708 | 0.0210 |  |  |  |  |  |
| 15 | 9 | 0.0708 | 0.0276 |  |  |  |  |  |
| 15 | 6 | 0.0501 | 0.0315 |  |  |  |  |  |
| 20 | 2 | 0.0537 | 0.0333 |  |  |  |  |  |
| 20 | 18 | 0.0537 | 0.0385 |  |  |  |  |  |
| 12 | 8 | 0.0935 | 0.0437 |  |  |  |  |  |
| 19 | 9 | 0.0708 | 0.0461 |  |  |  |  |  |
| 18 | 14 | 0.0501 | 0.0464 |  |  |  |  |  |
| 20 | 1 | 0.0617 | 0.0478 |  |  |  |  |  |
| 20 | 11 | 0.0708 | 0.0484 |  |  |  |  |  |
| 19 | 12 | 0.0617 | 0.0494 |  |  |  |  |  |

Table 4. Significant within-brain synchronisation (WBS) obtained from deoxy-Hb data for cooperative play (COOP) > independent play (IND) (left) and for COOP < IND (right). FDR-corrected (*Q* < 0.05).

| COOP > IND | | | |  | COOP < IND | | | |
| --- | --- | --- | --- | --- | --- | --- | --- | --- |
| *CHA* | *CHB* | *Freq (Hz)* | *Q value* |  | *CHA* | *CHB* | *Freq (Hz)* | *Q value* |
| 5 | 2 | 0.0935 | 0.0081 |  | 19 | 17 | 0.0537 | 0.0012 |
| 11 | 4 | 0.0935 | 0.0245 |  | 17 | 13 | 0.0308 | 0.0030 |
| 9 | 4 | 0.0935 | 0.0251 |  | 19 | 4 | 0.0380 | 0.0031 |
|  |  |  |  |  | 19 | 14 | 0.0436 | 0.0031 |
|  |  |  |  |  | 20 | 1 | 0.0331 | 0.0037 |
|  |  |  |  |  | 19 | 6 | 0.0407 | 0.0051 |
|  |  |  |  |  | 17 | 1 | 0.0308 | 0.0060 |
|  |  |  |  |  | 20 | 14 | 0.0467 | 0.0074 |
|  |  |  |  |  | 20 | 13 | 0.0331 | 0.0076 |
|  |  |  |  |  | 17 | 5 | 0.0380 | 0.0076 |
|  |  |  |  |  | 10 | 5 | 0.0617 | 0.0082 |
|  |  |  |  |  | 17 | 10 | 0.0354 | 0.0106 |
|  |  |  |  |  | 19 | 13 | 0.0407 | 0.0124 |
|  |  |  |  |  | 16 | 14 | 0.0467 | 0.0135 |
|  |  |  |  |  | 19 | 10 | 0.0331 | 0.0146 |
|  |  |  |  |  | 12 | 2 | 0.0407 | 0.0158 |
|  |  |  |  |  | 16 | 13 | 0.0501 | 0.0161 |
|  |  |  |  |  | 19 | 9 | 0.0354 | 0.0173 |
|  |  |  |  |  | 7 | 5 | 0.0380 | 0.0173 |
|  |  |  |  |  | 12 | 5 | 0.0308 | 0.0175 |
|  |  |  |  |  | 17 | 16 | 0.0501 | 0.0175 |
|  |  |  |  |  | 20 | 15 | 0.0436 | 0.0177 |
|  |  |  |  |  | 10 | 2 | 0.0501 | 0.0186 |
|  |  |  |  |  | 20 | 17 | 0.0308 | 0.0211 |
|  |  |  |  |  | 18 | 17 | 0.0308 | 0.0236 |
|  |  |  |  |  | 20 | 3 | 0.0380 | 0.0241 |
|  |  |  |  |  | 17 | 8 | 0.0380 | 0.0253 |
|  |  |  |  |  | 19 | 2 | 0.0407 | 0.0261 |
|  |  |  |  |  | 15 | 3 | 0.0308 | 0.0288 |
|  |  |  |  |  | 16 | 2 | 0.0467 | 0.0294 |
|  |  |  |  |  | 14 | 7 | 0.0407 | 0.0300 |
| Table 4 (continued) | | | | | | | | |
| COOP > IND | | | |  | COOP < IND | | | |
| *CHA* | *CHB* | *Freq (Hz)* | *Q value* |  | *CHA* | *CHB* | *Freq (Hz)* | *Q value* |
|  |  |  |  |  | 17 | 9 | 0.0407 | 0.0300 |
|  |  |  |  |  | 14 | 5 | 0.0407 | 0.0319 |
|  |  |  |  |  | 19 | 15 | 0.0380 | 0.0324 |
|  |  |  |  |  | 20 | 9 | 0.0407 | 0.0328 |
|  |  |  |  |  | 19 | 3 | 0.0380 | 0.0328 |
|  |  |  |  |  | 19 | 7 | 0.0354 | 0.0337 |
|  |  |  |  |  | 17 | 7 | 0.0407 | 0.0339 |
|  |  |  |  |  | 11 | 8 | 0.0935 | 0.0403 |
|  |  |  |  |  | 17 | 6 | 0.0407 | 0.0409 |
|  |  |  |  |  | 16 | 4 | 0.0308 | 0.0411 |
|  |  |  |  |  | 18 | 1 | 0.0575 | 0.0417 |
|  |  |  |  |  | 19 | 8 | 0.0380 | 0.0437 |
|  |  |  |  |  | 19 | 5 | 0.0661 | 0.0462 |
|  |  |  |  |  | 7 | 2 | 0.0501 | 0.0478 |
|  |  |  |  |  | 5 | 1 | 0.0537 | 0.0479 |
|  |  |  |  |  | 19 | 1 | 0.0354 | 0.0484 |
|  |  |  |  |  | 16 | 1 | 0.0575 | 0.0498 |
